# Determinants of access to insecticide-treated nets in Sub-Saharan Africa: A multilevel cross-country analysis using 29 DHS data

**DOI:** 10.1371/journal.pone.0330431

**Published:** 2025-09-10

**Authors:** Jember Azanaw, Eshetu Abera Worede

**Affiliations:** Department of Environmental and Occupational Health and Safety, Institute of Public Health, College of Medicine and Health Sciences, University of Gondar, Gondar, Ethiopia; Freelance Consultant, Myanmar, MYANMAR

## Abstract

**Background:**

Having access to Insecticide-Treated Nets (ITNs) is crucial for avoiding malaria in Sub-Saharan Africa (SSA), where the disease burden is disproportionately high. Despite their efficacy, socioeconomic, demographic, and geographic factors continue to cause notable differences in ITN access within and between nations. By employing a multilevel analysis of data from 29 Demographic and Health Surveys (DHS) throughout SSA, this study seeks to fill knowledge gaps about the factors that influence access at the individual and community levels.

**Methods:**

The study utilized data from 29 DHS surveys in SSA, which encompassed 214,181 households. Factors affecting access to ITN at the individual and community levels were examined using multilevel logistic regression models. Household head characteristics (sex, marital status, education, wealth index, television access, and family size) were among the individual-level factors. On the other hand, community-level characteristics included geography, kind of home, poverty, media exposure, and education. The Akaike Information Criterion (AIC), Bayesian Information Criterion (BIC), and deviance were used to evaluate the model’s fitness. Measures of variation, including the Median Odds Ratio (MOR), Proportional Change in Variance (PCV), and Intraclass Correlation Coefficient (ICC), were used to assess the effects at the community level.

**Results:**

ITNs were reported by just 32.11% of households, with notable differences across socioeconomic classes and geographical areas. Higher ITN access was substantially correlated with married household heads, wealthier families, larger family sizes, and higher educational attainment. Access was also greatly enhanced by community-level factors such as urban residency, media exposure, and higher education. The best fit was Model 3, which explained 15.24% of the variance in ITN access by combining characteristics at the person and community levels. There were clear regional differences, with West Africa having higher probabilities of access than East Africa (AOR = 4.48).

**Conclusion:**

The study highlights the multifaceted determinants of ITN access in SSA, emphasizing the need for targeted interventions addressing both individual and community-level barriers. Strengthening distribution networks, increasing funding for subsidized ITN programs and enhancing public health education is essential for achieving universal coverage and reducing the malaria burden in the region.

## Background

In Sub-Saharan Africa (SSA), a region that is disproportionately affected by malaria, having access to insecticidal nets (ITNs) is essential to preventing the disease [1,2] {Scates, 2021 #371}. By acting as a physical and chemical barrier against mosquitoes, ITNs effectively reduce the spread of malaria; yet, notable differences in access still exist between nations and within communities [3,4]. According to global health initiatives like the Roll Back Malaria Partnership and the Sustainable Development Goals (SDGs), attaining universal coverage and lowering malaria-related morbidity and mortality depend on an understanding of the factors influencing ITN access. Nonetheless, the World Health Organization (WHO) stated in 2023 that approximately 249 million cases of malaria, or 58 cases per 1000 population risk, occurred in 85 malaria-endemic countries in 2022 [5]. A complicated interaction between socioeconomic, demographic, and geographic factors exacerbates the prevalence of malaria in SSA [6,7]. According to WHO 2019, African regions recorded an estimated 215 million diseases of malaria, accounting for about 94% of all cases [8]. Poverty limits individuals’ ability to acquire ITNs, while rural communities often face logistical challenges in distribution [9,10]. The size of the household, educational attainment, and knowledge of malaria prevention techniques are important factors that influence ITN ownership and use [11–14]. National policies and international donor support further shape ITN access, yet inconsistent implementation of strategies leaves vulnerable populations unprotected.

Climate change is an emerging driver of malaria transmission in SSA, as rising temperatures and altered rainfall patterns expand mosquito habitats and extend transmission seasons [15,16]. Developing countries, including many in SSA, are particularly vulnerable to these effects due to limited healthcare infrastructure and resources to adapt to shifting disease patterns [17–19]. This amplifies the need for equitable ITN distribution to mitigate the growing malaria risk associated with climate variability.

According to studies findings, the gender of the household head, Marital status, and age of the household head [20] wealth index, family size, educational status [21–23]. However, these studies often focused on single-country contexts or lacked the analytical depth to account for individual and community-level factors influencing ITN access [12,13,24,25]. Additionally, they usually failed to consider the wider environmental and sociopolitical aspects, such as how climate change affects the dynamics of malaria transmission. This work fills important knowledge gaps about ITN that obstruct successful malaria prevention measures by incorporating discrepancies at several levels and merging diverse data sources to provide a more thorough picture of the structural and systemic impediments to ITN access. In light of these difficulties, a multilevel examination of data from 29 Demographic and Health Surveys (DHS) offers a special chance to pinpoint important factors influencing ITN availability in various settings. By identifying the fundamental factors that contribute to ITNs in Africa, such an analysis can help guide focused actions. To improve malaria control efforts in the face of environmental and socioeconomic challenges, it is imperative to address these drivers in order to close gaps in ITN coverage

## Method

### Study area and data source

This study combines information from the 29 SSA Demographic and Health Surveys to examine health outcomes associated with insecticidal-treated nets in different geographical areas. Because the study’s regions include South Africa, East Africa, Central Africa, and West Africa, a thorough examination of regional and socioeconomic differences is possible. In 2015–2016, Angola, Malawi, and Tanzania conducted surveys; recent survey data are also available for Benin (2017/2018), Burundi (2016/2017), Cameroon (2018), and the Democratic Republic of the Congo (2013/2014). Other nations with survey years are Ivory Coast (2011/2012), Burkina Faso (2010),.Ghana (2014), Gambia (2019/2020), Guinea (2018), Kenya (2014), Comoros (2012), Liberia (2019/2020), Lesotho (2014), Mali (2018), Nigeria (2018), Namibia (2013), Rwanda (2019/2020), Sierra Leone (2019), Senegal (2019), Chad (2014/2015), Togo (2013/2014), Uganda (2016), South Africa (2016), Zambia (2018), Zimbabwe (2015), and Ethiopia (2016). Burkina Faso provided the oldest data in 2010, while Gambia, Liberia, and Rwanda provided the most recent data, which was collected in 2019/2020. DHS uses a two-stage cluster sampling technique to choose households. Cluster enumeration areas (EAs), which are usually blocks in urban areas or villages in rural regions, were sampled in the first stage using a probability proportionate to the population size technique. Following a listing of every home in the targeted area, 25–30 homes were randomly selected for interviews in the second round. To obtain a representative sample of houses, this sampling technique was applied. This study included 214,181 weighted samples in total. It uses a multistage stratified sample technique to gather population, health, and nutrition data that are nationally representative.

Both community-level and individual-level factors that influence health outcomes are covered in detail by the study. Understanding the factors that contribute to disease transmission in these areas requires knowledge of household demographics, educational attainment, media exposure, and access to basic healthcare and sanitation facilities. Trained field personnel use structured questionnaires that are uniform across nations to guarantee comparability when conducting in-person interviews. Variations at the individual and community levels can be taken into account by using multilevel models, which shed light on the hierarchical nature of health data.

### Study variables

#### Individual-level factors.

With individual variables including the household head’s (HHH) sex, marital status, education level, wealth index, television access, and family size, the model incorporates both community-level and individual-level characteristics. It is anticipated that these individual-level determinants may have an impact on health outcomes, including illness prevalence. For instance, in health studies, sex (male or female) is usually a significant predictor. At the same time, socioeconomic characteristics that influence access to healthcare through ITN are captured by marital status (unmarried/married) and education level (no education, primary, secondary, and higher). Health behaviors and outcomes can also be influenced by lifestyle, income, and information access factors, such as family size (<4, 4–7, > 7), television access (yes/no), and wealth index (poor, middle, rich).

#### Community level factors.

Media exposure, poverty level, type of residence, and community education were community-level variables. Members of the same community or region are impacted by these community-level elements, which are combined at the group level. Certain community-level characteristics are derived from individual-level ones, where classification is done using mean values. To reduce bias and preserve the analysis’s robustness, the percentage of families with > 50% education is classified as high community-level education, and those with <50% education are classified as low community-level education. Using the mean value, the percentage of homes with community-level media exposure is divided into two categories: unexposed (those with ≥50%) and higher exposed (those with <50%). Poverty (poor, lower middle and upper middle income) is a reflection of economic circumstances that influence health disparities. Residential type (rural vs. urban) and area (East Africa, Central Africa, West Africa, South Africa) represent patterns of urbanization and geography that are frequently associated with access to health services and infrastructure.

#### Outcome variable.

“Has a mosquito bed net for sleeping in the household?” was a yes-or-no question that asked about the physical availability of insecticidal-treated nets. Those who answer “yes” are then classified as having access to insecticidal-treated nets, denoted by a 1, and those who answer “no” are denoted as not having access.

### Statistical analysis

Due to the hierarchical nature of the data, a multilevel logistic regression statistical approach was implemented. In this context, individual-level factors are nested within community-level factors. This model allows for analyzing both individual- and community-level predictors while accounting for the variability within and between communities. Random intercepts were included to capture the variance across communities, ensuring that the model accurately reflects the clustered nature of the data.

In the analysis, variables with a p-value of <0.2 in the bivariable analysis were included in the multivariable analysis to identify significant factors affecting ITN access. This study included individual-level variables such as sex and marital status of household heads, educational status, wealth index, television watching habits, and family size, as well as community-level variables, community education level, community-level media exposure, community-level poverty, type of residence, and region. Statistical significance in the multivariable analysis was assessed using adjusted odds ratios (AORs) with 95% confidence intervals (CIs). Variables with p-values <0.05 in the multivariable model were considered statistically significant, indicating their independent association with ITN access after controlling for other factors. This rigorous approach ensured that only robust predictors were identified while accounting for potential confounders. All statistical analysis were carried out using STATA version 17.

The following four nested models were developed to assess individual- and community-level factors determining ITN access, with each model progressively incorporating additional predictors.

Model 0, the null model, was constructed without any explanatory variables to estimate the baseline variance in ITN access attributable to differences between communities. This model provided a reference for assessing the contribution of subsequent individual- and community-level factors.


logit(Pij)=β0+uj

Where:

P_ij_: Probability of ITN access for individual i in community j

β0: Overall intercept

uj: Random effect at the community level

Model I, included individual-level factors (Marital status of the HHH, Educational status of the HHH, Wealth index, watches television, family size) to account for variations in ITN access.


$$\begin{array}{c} logit(Pij)=\beta 0+\beta 1(MaritalStatus)ij+\beta 2(EducationLevel)ij+\beta 3(WealthIndex)ij\\ \;\mathrm{-51pt}+\beta 4(MediaExposure)ij+\beta 5(HouseholdSize)ij+uj \end{array}$$

Where: β1 to β5: Coefficients for included individual-level predictors

In Model II, community-level predictors (Community-level education, Community-level media exposure, Community-level poverty, type of residence, Region) were added to know broader contextual factors affecting ITN access.


$$\begin{array}{cc} logit(Pij) & =\beta 0+\beta 6(CommunityEducation)j+\beta 7(CommunityMediaExposure)j\\ & +\beta 8(CommunityPoverty)j+\beta 9(TypeofResidence)j+\beta 10(Region)j+uj \end{array}$$

Where: β6 to β10: Coefficients for included community-level predictors

Model III incorporated both individual- and community-level factors to provide a comprehensive analysis of determinants influencing ITN access. This model tested the interaction and cumulative impact of predictors from both levels, capturing the full scope of influences affecting ITN accessibility.


$$\begin{array}{cc} logit(Pij) & =\beta 0+\beta 1(MaritalStatus)ij+\beta 2(EducationLevel)ij+\beta 3(WealthIndex)ij\\ & +\beta 4(MediaExposure)ij+\beta 5(HouseholdSize)ij+\beta 6(CommunityEducation)j\\ & +\beta 7(CommunityMediaExposure)j+\beta 8(CommunityPoverty)j\\ & +\beta 9(TypeofResidence)j+\beta 10(Region)j+uj \end{array}$$

### Measures of variations and model fitness

In multilevel modeling, measures of variation such as the Median Odds Ratio (MOR), Proportional Change in Variance (PCV), and Intraclass Correlation Coefficient (ICC) are essential for understanding the proportion of variance attributed to group-level (community) versus individual-level factors. The MOR quantifies the odds of an individual having a higher outcome if they were to switch between two randomly selected groups, providing insights into the magnitude of between-group variation. The PCV is used to assess the reduction in variance when individual-level predictors are included in the model, indicating how much of the variance is explained by these predictors. ICC measures the proportion of total variance in the outcome that is attributable to differences between groups, such as communities, relative to the total variance, including within-group (individual) variation. These measures help determine the importance of community-level factors in explaining health outcomes.


PCV=((σ_{u0}^2−σ_{u1}^2))/(σ_{u0}^2)

Where:

$\sigma_{\left\{u0\right\}}^{2}=$Variance of random effects from the null model (without predictors).

$\sigma_{\left\{u1\right\}}^{2}$= Variance of random effects from the model with predictors included.

PCV = Proportional Change in Variance


$$MOR=\text{e}x\text{p}\left(\sqrt{}2\cdot \sigma u\right)$$

Where: MOR = Median Odds Ratio


ICC=(σ_u^2)/(σ_u^2+π^2/3)

Where: ICC = Intraclass Correlation

Statistics such as the Akaike Information Criterion (AIC), Bayesian Information Criterion (BIC), and Deviance are used to assess model fitness in addition to the measures of variance. These tests evaluate the model’s complexity and fit. By compensating for model complexity, the AIC is used to compare the relative goodness-of-fit of several models; a lower AIC denotes a better-fitting model. In a similar vein, BIC penalizes model complexity as well, but the penalty gets higher as sample size grows. When evaluating several model parameters, both AIC and BIC help choose a model. Higher-fitting models are shown by lower deviance values, which are a measure of model fit and represent the difference between observed and projected values. By balancing explanatory power and model simplicity, these model fitness tests assist in guaranteeing that the model not only adequately explains the data but also stays clear of overfitting.

### Ethics approval and consent to participate

With previous clearance and consent from the DHS Program, the study was carried out using publicly available Demographic and Health Survey (DHS) data. Ethics approval and consent to participate are not applicable (because it is secondary data). However, during primary data collection by DHS participants, anonymity was protected, and the ethical requirements were adhered to. The following website accessed the data.

https://dhsprogram.com/data/dataset_admin/login_main.cfm?CFID=10818526&CFTOKEN=c131014a480fe56-4E0C6B7F-F551-E6B2-50.

## Results

### Socio-demographic characteristics

The survey includes 214,181 weighted households in total. The majority of families in Sub-Saharan Africa (46.69%) lacked access to electricity. Just 11.03% of the remaining households were classified as upper-middle-income, while the remaining households were classified as low-income (59.37%) and lower-middle-income (33.45%). Information from four Sub-Saharan African regions is included in the dataset: South Africa, with 3.91% of households, West Africa, with 42.15%, Central Africa, with 10.13%, and East Africa, with 43.80% of families (Table 1).

**Table 1:** Socio-demographic characteristics at the individual and community levels among households in Sub-Saharan African countries (N = 214,181).

Variables	Insecticidal Treated Net Availability	
	No n (%)	Yes n (%)
**Sex of HHH**		
Male	48,201 (22.50)	108,779 (50.79)
Female	20,583 (9.61)	36,618 (17.10)
**Age of HHH in year**		
<35	23,376 (10.91)	47,944 (22.38)
35–60	32,443 (15.15)	72,055 (33.64)
>60	12,965 (6.05)	25,398 (11.86)
**Educational Status of HHH (n = 214,080)**		
No education	22,768 (10.64)	46,952 (21.93)
Primary	22,101 (10.32)	46,940 (21.93)
Secondary	17,969 (8.39)	37,340 (17.44)
Higher	5,904 (2.76)	14,106 (6.59)
**Marital status of HHH (n = 208,750)**		
Unmarried	22,719 (10.88)	31,463 (15.07)
Married	44,139 (21.14)	110,429 (52.90)
**Family size**		
< 4	29,009 (13.54)	44,662 (20.85)
4–7	31,217 (14.58)	74,353 (34.72)
>7	8,558 (4.00)	26,382 (12.32)
**Accessibility of Electricity**		
Yes	26,925 (12.57)	87,257 (40.74)
No	41,859 (19.54)	58,140 (27.15)
**Watching Television**		
No	46,258 (21.60)	95,749 (44.71)
Yes	22,524 (10.52)	49,635 (23.18)
**Wealth index**		
Poor	26,164 (12.22)	2,779 (24.13)
Middle	13,245 (6.18)	28,882 (13.48)
Rich	29,375 (13.72)	64,842 (30.27)
**Residence**		
Rural	40,990 (19.14)	91,213 (42.59)
Urban	27,794 (12.98)	54,184 (25.30)
**Community level Income (n = 195,875)**		
Low income	34,634 (17.68)	81,663 (41.69)
Lower middle income	20,925 (10.68)	44,600 (22.77)
Upper middle income	8,669 (7.31)	5,384 (3.72)
**Community level education**		
Low	32,732 (15.28)	73,536 (34.33)
High	36,052 (16.83)	71,861 (33.55)
**Community-level media exposure**		
Exposed	80,894 (34.66)	6,044 (2.59)
Unexposed	29,734 (13.88)	51,224 (23.92)
**Region**		
East Africa	29,781 (13.90)	64,038 (29.90)
Central Africa	7,108 (3.32)	14,596 (6.81)
West Africa	26,429 (12.34)	63,849 (29.81)
Southern Africa	5,466 (2.55)	2,914 (1.36)

### Prevalence of insecticide-treated nets in Sub-Saharan Africa

In Sub-Saharan Africa, 32.11% (68,784/214,181) of households reported having insecticide-treated nets, while 67.89% (145,397/ 214,181) did not.

### Predictors of insecticide-treated nets access

This stepwise modeling approach highlights the incremental value of adding contextual and regional factors beyond individual characteristics, enabling a clearer understanding of the relative importance and stability of predictors across models. The change in adjusted odds ratios (AORs) and variance inflation factors (VIFs) across the models further demonstrates the evolving influence and interaction of predictors in explaining the outcome.

The household head’s (HHH) marital status, educational attainment, wealth index, television viewing, and number of family members were among the individual-level factors, according to the final model, while the community-level factors, including type of residence, poverty, media exposure, and education, were statistically significant.

Married household heads had a significantly higher likelihood of ITN access, with an AOR of 1.61 (95% CI: 1.56, 1.65), compared to unmarried heads.

Higher educated household heads were most likely to have access to ITN, with an AOR of 12.56 (95% CI: 12.22, 12.91). The AOR for those with secondary education was 12.98 (95% CI: 12.59, 13.38), but the AOR for those with primary education was 1.43 (95% CI: 1.37, 1.49), which represented a smaller but still substantial increase in access. The reference group was heads of households with no formal education.

Wealthier households were more likely to access ITNs. The AOR for rich households was 12.46 (95% CI: 11.34, 13.70), and for middle-income households, it was 1.16 (95% CI: 1.13, 1.20), compared to poor households.

The odds of ITN access were significantly higher for households with watching television, with an AOR of 1.12 (95% CI: 1.09, 1.15), compared to those without access to television.

Households with larger family sizes had a higher likelihood of ITN access. For families with 4–7 members, the AOR was 1.92 (95% CI: 1.84, 2.00), and for those with more than seven members, it was 1.48 (95% CI: 1.43, 1.53), compared to households with fewer than four members.

Communities with higher education levels had a significantly higher likelihood of ITN access, with an AOR of 14.72 (95% CI: 13.32, 16.28), compared to communities with lower education levels.

Community level media exposure was more likely to have ITN access, with an AOR of 1.39 (95% CI: 1.30, 1.49), compared to those with no media exposure.

Higher-income communities were associated with higher access to ITNs. The AOR for upper middle-income communities was 5.04 (95% CI: 4.69, 5.42), and for lower middle-income communities, it was 5.32 (95% CI: 4.97, 5.70), compared to low-income communities.

Urban areas had significantly higher odds of ITN access, with an AOR of 3.88 (95% CI: 3.68, 4.09), compared to rural areas.

Regional differences were evident in ITN access. Households in Central Africa had an AOR of 3.63 (95% CI: 3.44, 3.83), in West Africa, the AOR was 4.48 (95% CI: 4.26, 4.70), and in South Africa, the AOR was 3.48 (95% CI: 3.32, 3.66), compared to East Africa (Table 2).

**Table 2:** Multilevel logistic regression modeling for both individual and community-level factors.

Variables	Model 0 (Null model)	Model 1 AOR (95% CI)	Model 2 AOR (95% CI)	Model 3 AOR (95% CI)
**Individual-level Factors**				
**Sex of HHH**				
Female		1.84 (1.80, 1.88)**		0.91 (0.89, 1.14)
Male		1		1
**Marital status of HHH**				
Unmarried		1		1
Married		1.84(1.80,1.88)**		1.61(1.56, 1.65)**
**Educational status of HHH**				
Higher		6.044(6.0210, 6.0693)**		12.56(12.22,12.91)**
Secondary		4.0356(4.0105, 4.0613)*		12.98(12.59,13.38)**
Primary		1.1928(1.1518, 1.2353)**		1.43(1.37,1.49)**
No education		1		1
**Wealth index**				
Rich		1.1627(1.1325, 1.1937)**		12.46(11.34,13.70)**
Middle		1.1697(1.1449, 1.1951)**		1.16(1.13,1.20)**
Poor		1		1
**Watching television**				
Yes		1.1483(1.1252, 1.1719)**		1.1189(1.0855, 1.1533)**
No		1		1
**Number of family**				
<4		1		1
4–7		1.5216,1.4913, 1.5525)**		1.92(1.84,2.00)**
>7		1.9018(1.8475, 1.9577)**		1.48(1.43,1.53)**
**Community-level factors**				
**Community-level education**				
Low			1	1
Higher			13.36(12.19,14.65)**	14.72(13.32,16.28)**
**Community-level media exposure**				
Unexposed			1	1
Exposed			13.89(12.86,15.01)**	1.39(1.30,1.49)**
**Community-level poverty**				
Upper middle income			3.89(3.75, 4.04)**	5.04(4.69, 5.42)**
Lower middle income			4.49(4.32,4.68)**	5.32(4.97,5.70)**
Low income			1	1
**Type of residence**				
Rural			1	1
Urban			11.36(11.14,11.69)**	3.88(3.68,4.09)**
**Region**				
South Africa			3.89(3.70,4.08)**	3.48(3.32,3.66)**
Central Africa			3.96(3.75,4.18)**	3.63(3.44,3.83)**
West Africa			5.11(4.86,5.35)**	4.48(4.26,4.70)**
East Africa			1	1
VIF		1.50	2.43	4.68

**Key:** 1 = reference, **P-value < 0.001(Adjusted OR), *P-value < 0.05 (Adjusted OR), HHH = household head, HH = household, Model 0 (Null model) fitted without predictor variables; Model I adjusted for individual-level variables; Model II adjusted for community-level variables; Model III the final model adjusted for the individual- and community-level predictors.

### Measures of variation and model fitness statistics

Indicating variations in the percentage of variability in ITN access among the models, the variance values for the models ranged from 0.4676 to 0.5775. With a range of 2.26 to 2.76, the Model of Relative Risk (MOR) indicated different levels of variation at the community level. With values ranging from 8.98% to 15.24%, the Proportion of Variance Explained (PCV) demonstrated a notable improvement, highlighting the role that both individual and community-level factors play in the overall model. The Intra-Class Correlation (ICC) values, which show the percentage of overall variability attributable to the community level, ranged from 12.40% to 14.93%.In terms of model fitness, the Akaike Information Criterion (AIC) values for each model improved with each new model, falling from 270,502.70 to 228,977.00. As the model complexity rose, the Bayesian Information Criterion (BIC) values also decreased, with the lowest BIC being 229,180.10. This suggests that the model is more efficient. The addition of more components improved the model fit, as evidenced by the Deviance values, which likewise steadily decreased from 270,498.68 to 228,936.96 (Table 3).

**Table 3:** Measures of variations and model fitness test statistics in LLMNs.

Measures of variation				
Metrics	Model 0 (Null model)	Model 1	Model 2	Model 3
Variance	0.4676052	0.5137629	0.4895376	0.5775379
MOR	2.63	2.76	2.69	2.26
PCV	Ref.	8.98%	4.715%	15.24%
ICC	12.40%	13.50%	12.95%	14.93%
**Model fitness test statistics**				
AIC	270502.70	257112.50	242276.80	228977.00
BIC	270523.20	257235.50	242348.10	229180.10
Deviance	270,498.68	257,088.54	242,262.76	228,936.96

**Key:** AIC = Akaike’s information criteria, BIC = Bayesian information criteria.

## Discussion

Sub-Saharan Africa’s access to insecticidal-treated net (ITN) prevalence data shows notable gaps in household access, which presents difficulties for the region’s malaria prevention initiatives. Of households, only 32.11% (31.20%, 32.98%) reported having access to ITNs for preventing malaria. This finding was lower than the result of the studies done in Ghana (57.4%) [25] and Uganda (86%) [26]. However, this result was higher than the study’s finding in Myanmar, where 22.3% of participants had access to ITNs [27].

Logistical issues in distribution, particularly in remote and rural areas, hinder access, while economic barriers prevent households from purchasing nets independently. Furthermore, public health campaigns to raise awareness about the importance of ITNs may be insufficient or fail to reach marginalized populations. Inequities in access often correlate with socio-economic and demographic variables, including poverty, household size, education levels, and gender dynamics in decision-making [28].

The lack of ITNs of households raises concerns about malaria vulnerability, particularly in regions where transmission rates are high. This gap leaves millions unprotected against mosquito bites, increasing the risk of infection and its associated health and economic burdens. Specifically, children under five and pregnant women, who are among the most vulnerable populations, face heightened exposure due to the inadequate availability of ITNs in many households [29].

### Measures of variation and model fitness

Model 0, the null model, has a variance of 0.4676, serving as the baseline. The introduction of predictors in Model 1 increases the variance to 0.5138, suggesting that the additional variables capture more variability in ITN access. However, subsequent models slightly reduce the variance, with Model 3 showing the highest variance (0.5775), implying that it accounts for the most community and individual-level determinants.

The null model (MOR = 2.63) demonstrates moderate heterogeneity. This value increases slightly in Model 1 (2.76) and Model 2 (2.69), reflecting the inclusion of significant predictors but also highlighting persistent unexplained heterogeneity. Model 3 shows a notable decrease in MOR (2.26), indicating that this model reduces between-group disparities more effectively than earlier iterations.

Model 1 achieves an 8.98% variance reduction, while Model 2 achieves only a 4.72% reduction, suggesting that the additional variables in Model 2 have limited explanatory power. However, Model 3 shows the highest PCV (15.24%), demonstrating its superior capability in capturing the primary variation in ITN access.

ICC indicates the proportion of total variation attributable to between-group differences. The null model shows an ICC of 12.40%, emphasizing significant between-group variability. ICC slightly increases in Model 1 (13.50%) and Model 2 (12.95%), but Model 3 reaches the highest ICC (14.93%), signifying that this model captures more contextual and group-level determinants.

Model fitness is assessed using the Akaike Information Criterion (AIC), Bayesian Information Criterion (BIC), and deviance, with lower values indicating better fit. AIC and BIC values decrease progressively from Model 0 to Model 3, indicating improved model performance. Specifically, AIC declines from 270,502.70 in Model 0–228,977.00 in Model 3, while BIC decreases from 270,523.20 to 229,180.10. Similarly, deviance, reflecting the lack of fit, drops significantly across models, with Model 3 achieving the lowest deviance (228,936.96), highlighting its superior fit to the data. The analysis demonstrates that Model 3 provides the best explanation for variations in ITN access, effectively balancing individual- and group-level determinants while improving model fit.

### Determinants of long-lasting mosquito nets

#### Individual-level predictors.

Married household heads demonstrated significantly higher odds of accessing insecticide-treated nets (ITNs) compared to their unmarried counterparts, which likely reflects several interconnected social and economic factors. Marriage often brings greater household stability and shared decision-making, enabling more effective prioritization and allocation of resources toward essential health needs such as malaria prevention tools. The collaborative nature of married couples can facilitate joint planning and budgeting, increasing the likelihood of investing in protective measures like ITNs. Additionally, married individuals may benefit from combined or higher household incomes, which enhances their financial capacity to obtain ITNs either through purchase or by fulfilling criteria for distribution programs. Furthermore, marriage may expand social support networks, providing access to information, assistance, and communal resources that facilitate ITN acquisition and usage. Altogether, these factors suggest that marital status is an important determinant influencing the accessibility and utilization of malaria prevention interventions within households.. The result is in line with similar studies conducted in different parts of the world [30,31] and contradicted by other research done in Cameron [32].

The findings indicate a strong positive association between the educational level of household heads and the likelihood of accessing insecticide-treated nets (ITNs), with adjusted odds ratios (AORs) significantly higher for those with secondary and higher education compared to those with no education. This suggests that as the level of education increases, so does the understanding and awareness of malaria prevention strategies, leading to proactive health-seeking behaviors such as acquiring and using ITNs. Education likely enhances comprehension of disease transmission, the importance of prevention, and the proper use of ITNs, making educated individuals more likely to take preventive actions. Even a basic level of formal education, such as primary schooling, contributes positively to ITN access, although to a lesser extent, as evidenced by the AOR of 1.43 for those with primary education. This trend underscores the crucial role of educational attainment in promoting public health interventions and suggests that expanding educational opportunities could indirectly enhance the uptake of malaria prevention measures like ITN usage. This finding is consistent with previous studies [14,33,34] and contradicts the study done in Ghana [35].

Economic status is the other critical determinant of access to health resources, including ITNs [31,36]. In this finding, the rich and middle class had odds compared to poor households. The ability of wealthy households to get, maintain, and purchase insecticidal nets—as well as perhaps better access to health information and resources—is probably the reason for this substantial correlation. Socioeconomic differences in malaria prevention coverage are highlighted by the fact that poor households, the reference group, are the ones with the lowest likelihood of owning an ITN. This finding is supported by previous similar studies [25,27,37] but contradicted by other studies conducted in Myanmar [38,39] and Ghana [35]. Variations in study settings, study period demographic characteristics, and implementation tactics may be the cause of discrepancies in ITN findings between studies. ITN access and use can be strongly impacted by variations in the infrastructure of the health system, the time of data collection, and the degree of malaria transmission. Furthermore, the effectiveness and uptake of ITN distribution may be affected by socioeconomic and cultural factors such as household preferences, income, and education [40,41]. Households that watched television had higher odds of accessing ITNs. Media exposure likely increases awareness of malaria prevention measures. Possible explanation: households with television access are more exposed to public health messaging, enabling them to recognize the importance of ITNs. The effect of watching television is also shown in a previous study done in Nepal [42].

The findings revealed that households with larger family sizes were more likely to have access to insecticide-treated nets (ITNs), with those comprising 4–7 members and more than 7 members showing significantly higher odds of ITN access compared to smaller households with fewer than 4 members. This pattern may be attributed to targeted distribution strategies that prioritize larger families under the assumption that they have greater needs, leading to a higher likelihood of receiving nets during public health campaigns. However, while such strategies may increase the presence of ITNs at the household level, they do not necessarily ensure equitable access among all members. In larger families, the number of ITNs may be insufficient relative to the number of individuals, potentially resulting in inadequate coverage and unequal protection, particularly for more vulnerable members such as children under five or pregnant women. Thus, despite improved access at the household level, challenges in intra-household allocation may compromise the overall effectiveness of ITN interventions in larger families. This finding is in line with earlier studies [30,43,44].

Communities with higher education levels exhibit significantly greater odds of accessing insecticide-treated nets (ITNs) compared to those with lower education levels, highlighting the critical influence of collective educational attainment on health-related behaviors and outcomes. This association likely stems from the fact that educated communities tend to cultivate a stronger culture of health awareness, where knowledge about disease prevention and the benefits of interventions like ITNs is more widely disseminated and valued. Higher education facilitates better understanding of malaria transmission and the importance of preventive measures, which in turn promotes community advocacy for access to such resources. Additionally, educated populations are more likely to engage with health programs, demand accountability from service providers, and effectively utilize available health infrastructure. Collectively, these factors contribute to improved mobilization and utilization of malaria-preventive tools, underscoring the pivotal role of community-level education not only in individual behavior change but also in fostering an environment that supports sustained public health gains [45].This result was supported by previous studies [46–49].

Communities with greater media exposure demonstrated significantly higher odds of accessing insecticide-treated nets (ITNs), highlighting the influential role that media can play in malaria prevention efforts. Media campaigns serve as powerful tools to disseminate vital health information, increase awareness about the benefits of using ITNs, and educate the public on correct usage and maintenance. By consistently reaching broad audiences, these campaigns can shape positive health behaviors, reduce misconceptions, and motivate individuals and households to prioritize preventive measures. Moreover, community-level media interventions can create a supportive environment that normalizes ITN use, thereby amplifying demand and encouraging widespread uptake. This underscores the importance of integrating well-designed, culturally appropriate media strategies into malaria control programs to enhance coverage, improve public knowledge, and ultimately reduce malaria transmission [50].

Communities classified as upper-middle income demonstrated significantly higher odds of accessing insecticide-treated nets (ITNs) compared to their low-income counterparts, underscoring the critical role of economic disparities in shaping health resource accessibility. This disparity likely stems from the fact that higher-income communities are better positioned to invest in and sustain health-related infrastructure, including efficient distribution channels for preventive tools like ITNs. Economic development at the community level not only enhances purchasing power and access to information but also facilitates stronger institutional support for public health initiatives. Consequently, wealthier communities are more likely to benefit from consistent availability and use of ITNs, which are essential for malaria prevention. In contrast, low-income communities may face structural barriers such as inadequate distribution systems, poor transportation networks, and limited awareness, all of which hinder equitable access to these life-saving resources [45].

The observed higher odds of accessing insecticide-treated nets (ITNs) in urban areas compared to rural areas can be attributed to the persistent urban-rural divide in resource allocation and infrastructural development. Urban settings generally benefit from well-established distribution networks, more efficient healthcare delivery systems, and greater exposure to public health awareness campaigns, all of which facilitate easier access to preventive health interventions like ITNs. In contrast, rural communities often face substantial barriers, including poor road infrastructure, limited healthcare facilities, and lower health literacy, which hinder the timely and equitable distribution of ITNs. Additionally, logistical challenges in reaching remote or geographically isolated areas may delay or disrupt ITN supply chains, further contributing to the disparity in access between urban and rural populations [51]. This was consistent with previous studies conducted in Zimbabwe [52] and Nepal [42].

The observed regional differences in the odds of accessing insecticide-treated nets (ITNs) reveal that households in West Africa had the highest likelihood of access, followed by those in Central and Southern Africa, when compared to East Africa. These disparities likely reflect a combination of factors, including variations in malaria transmission intensity, with West and Central Africa historically bearing a higher malaria burden, thereby prompting intensified intervention efforts. National health policies across these regions may differ in their commitment and resource allocation to malaria prevention, with some governments exhibiting stronger political will and leadership in implementing ITN distribution programs. Additionally, external donor support, such as from the Global Fund and international NGOs, may be more substantial or better coordinated in certain regions, enhancing access. Effective logistical strategies for distributing ITNs, including timely mass campaigns and integration with routine health services, alongside regional prioritization in malaria control strategies, may further explain the higher coverage. Collectively, these elements highlight how both epidemiological need and institutional capacity shape disparities in ITN access across African regions.

### Limitations

The first limitation, data on ITN access and other variables are self-reported, which may introduce recall or social desirability bias. The second limitation of the study does not account for cultural beliefs or behaviors that may influence ITN use, such as misconceptions about malaria or preferences for alternative prevention methods. The third limitation countries not included in the DHS surveys are excluded, which may limit the generalizability of the findings. The fourth limitation DHS data may not reflect recent changes in ITN distribution programs or malaria prevention efforts due to the inclusion of old DHS data. The fifth limitation of the study not account for health facility proximity, potential confounders, such as political instability or climate factors, which could influence ITN access. The sixth limitation is that the study could not assess temporal relationships due to the cross-sectional.

### Recommendations for future research

Incorporate qualitative methods to explore cultural, behavioral, and health system factors influencing ITN use. Include metrics on ITN usage and adequacy, not just ownership, to better assess the effectiveness of distribution programs. Involve community leaders and members in ITN distribution and education campaigns to address cultural barriers and increase acceptance. Use geospatial data to identify hotspots of low ITN access and target interventions more effectively.

## Conclusion

Addressing these disparities requires a multifaceted approach, including strengthening distribution networks, increasing funding for free or subsidized ITN programs, and improving public health education campaigns. The study emphasizes the multifaceted nature of ITN access in Sub-Saharan Africa, influenced by both individual and community-level factors. Addressing these determinants requires a holistic approach that combines education, economic development, and targeted public health interventions to improve access and reduce malaria burden in the region. This will not only help reduce the prevalence of malaria but also align with broader global health goals for equitable healthcare access in Sub-Saharan Africa.

## References

[1] Mwang’onde BJ. Beyond nets and sprays: transformative strategies for malaria resilience in Sub-Saharan Africa. Journal of Health & Biological Sciences. 2024;12(1):1–7.

[2] Scates S. Insecticide Treated Nets in Sub-Saharan Africa: Ownership, Access, Use, and Cost. Tulane University. 2021.

[3] Mehta J. Optimizing malaria control in Nigeria: A comprehensive review of LLIN effectiveness and policy frameworks. 2024.

[4] Ugwu FSO. Why the World Health Organization should reconsider long lasting insecticide nets (LLIN) and indoor residual spraying (IRS) in primary mosquito/malaria control in favour of house screening. Bio Research. 2023;21(1):1789–804. doi: 10.4314/br.v21i1.3

[5] Shin HI. 2023 World Malaria Report (Status of World Malaria in 2022). 2024.

[6] Villena OC, Arab A, Lippi CA, Ryan SJ, Johnson LR. Influence of environmental, geographic, socio-demographic, and epidemiological factors on presence of malaria at the community level in two continents. Sci Rep. 2024;14(1):16734. doi: 10.1038/s41598-024-67452-5 39030306 PMC11271557

[7] Padhi BK, Gaidhane AM, Satapathy P, Bushi G, Ballal S, Bansal P, et al. Assessing the impact of ecological, climatic, and socioeconomic factors on age-specific malaria incidence in India: a mixed-model approach using the Global Burden of Disease Study (2010-2019). Malar J. 2024;23(1):332. doi: 10.1186/s12936-024-05151-2 39516817 PMC11549856

[8] Organization WH. Malaria eradication: benefits, future scenarios and feasibility. World Health Organization. 2020.

[9] Sexton AR. Best practices for an insecticide-treated bed net distribution programme in sub-Saharan eastern Africa. Malar J. 2011;10:157. doi: 10.1186/1475-2875-10-157 21651815 PMC3121652

[10] Acosta D, Ludgate N, McKune SL, Russo S. Who has access to livestock vaccines? Using the social-ecological model and intersectionality frameworks to identify the social barriers to peste des petits ruminants vaccines in Karamoja, Uganda. Frontiers in Veterinary Science. 2022;9:831752. doi: 10.3389/fvets.2022.831752 35296060 PMC8918586

[11] Hunde OY, Hailu HE, Wondimu J, Dinku B, Ewnetu W. Assessment of long-lasting insecticide nets coverage, utilization, and associated factors among households in malaria elimination districts of Arsi Zone, Oromia Region, Ethiopia: A cross-sectional study. PLoS One. 2023;18(11):e0293728. doi: 10.1371/journal.pone.0293728 37917649 PMC10621819

[12] Ng’ang’a PN, Aduogo P, Mutero CM. Long lasting insecticidal mosquito nets (LLINs) ownership, use and coverage following mass distribution campaign in Lake Victoria basin, Western Kenya. BMC Public Health. 2021;21(1):1046. doi: 10.1186/s12889-021-11062-7 34078333 PMC8173981

[13] Aweis A, Salad AA, Araye FA, Ahmed AM, Wehlie OA, Osman AA, et al. Long-lasting insecticidal nets (LLINs) use among household members for protection against mosquito bite in Mogadishu districts. PLOS Glob Public Health. 2023;3(3):e0000724. doi: 10.1371/journal.pgph.0000724 36962968 PMC10019640

[14] Yeboah A. Comparative analysis of factors associated with insecticide-treated net utilization between rural and urban areas in Ghana: implication for malaria control and prevention. The University of Bergen. 2023.

[15] Leal Filho W, May J, May M, Nagy GJ. Climate change and malaria: some recent trends of malaria incidence rates and average annual temperature in selected sub-Saharan African countries from 2000 to 2018. Malar J. 2023;22(1):248. doi: 10.1186/s12936-023-04682-4 37641080 PMC10464074

[16] Semenza JC, Rocklöv J, Ebi KL. Climate Change and Cascading Risks from Infectious Disease. Infect Dis Ther. 2022;11(4):1371–90. doi: 10.1007/s40121-022-00647-3 35585385 PMC9334478

[17] Wright CY, Kapwata T, Naidoo N, Asante KP, Arku RE, Cissé G, et al. Climate Change and Human Health in Africa in Relation to Opportunities to Strengthen Mitigating Potential and Adaptive Capacity: Strategies to Inform an African “Brains Trust”. Ann Glob Health. 2024;90(1):7. doi: 10.5334/aogh.4260 38312714 PMC10836170

[18] Ulrich HH. How are health systems in sub-Saharan Africa adapting to protect human health from climate change threats? Institute of Tropical Medicine. 2023. 10.1016/S2542-5196(24)00075-5 38632905

[19] Oladipo HJ, Tajudeen YA, Oladunjoye IO, Yusuff SI, Yusuf RO, Oluwaseyi EM, et al. Increasing challenges of malaria control in sub-Saharan Africa: Priorities for public health research and policymakers. Ann Med Surg (Lond). 2022;81:104366. doi: 10.1016/j.amsu.2022.104366 36046715 PMC9421173

[20] Fokam EB, Kindzeka GF, Ngimuh L, Dzi KTJ, Wanji S. Determination of the predictive factors of long-lasting insecticide-treated net ownership and utilisation in the Bamenda Health District of Cameroon. BMC Public Health. 2017;17(1):263. doi: 10.1186/s12889-017-4155-5 28302093 PMC5356302

[21] Tassew A, Hopkins R, Deressa W. Factors influencing the ownership and utilization of long-lasting insecticidal nets for malaria prevention in Ethiopia. Malar J. 2017;16(1):262. doi: 10.1186/s12936-017-1907-8 28668084 PMC5493859

[22] Iornem TV, Abdulkardir SS, Akinrefon AA, Ornguga IG. Modelling of access to mosquito treated net in nigeria: using multilevel logistic regression approach. FJS. 2023;7(3):144–9. doi: 10.33003/fjs-2023-0703-1831

[23] Kawuki J, Donkor E, Gatasi G, Nuwabaine L. Mosquito bed net use and associated factors among pregnant women in Rwanda: a nationwide survey. BMC Pregnancy Childbirth. 2023;23(1):419. doi: 10.1186/s12884-023-05583-9 37280560 PMC10243234

[24] Njatosoa AF, Mattern C, Pourette D, Kesteman T, Rakotomanana E, Rahaivondrafahitra B, et al. Family, social and cultural determinants of long-lasting insecticidal net (LLIN) use in Madagascar: secondary analysis of three qualitative studies focused on children aged 5–15 years. Malaria Journal. 2021;20(1):168. doi: 10.1186/s12936-021-03705-2 33771162 PMC7995690

[25] Aheto JMK, Babah R, Dzokoto MK, Kwarah W, Alhassan Y. Predictors of mosquito bed net use among children under-fives in Ghana: a multilevel analysis of the 2019 malaria indicator survey. Malar J. 2023;22(1):196. doi: 10.1186/s12936-023-04634-y 37365602 PMC10291786

[26] Perkins JM, Krezanoski P, Takada S, Kakuhikire B, Batwala V, Tsai AC, et al. Social norms, misperceptions, and mosquito net use: a population-based, cross-sectional study in rural Uganda. Malar J. 2019;18(1):189. doi: 10.1186/s12936-019-2798-7 31159821 PMC6547474

[27] Win KM, Show KL, Sattabongkot J, Aung PL. Ownership and use of insecticide-treated nets in Myanmar: insights from a nationally representative demographic and health survey. Malar J. 2024;23(1):167. doi: 10.1186/s12936-024-04994-z 38807175 PMC11135007

[28] Andeskebtso AY, Ugochukwu NJ. Impact of socio-economic factors on women’s family planning decisions in Taraba State, Nigeria. Jalingo Journal of Social And Management Sciences. 2023;4(4):262–75.

[29] Kagaba AG. Socio-Economic Determinants and Malaria Risk: Assessing the Impact of Poverty, Housing Conditions, and Healthcare Accessibility in High-Incidence Regions. SCIENCES (NIJRMS). 2024;5(3).

[30] Diallo OO, Ozodiegwu ID, Camara A, Galatas B, Gerardin J. Factors associated with the ownership and use of insecticide-treated nets in Guinea: an analysis of the 2018 Demographic and Health Survey. Malar J. 2023;22(1):29. doi: 10.1186/s12936-023-04463-z 36703147 PMC9878948

[31] Pooseesod K, Parker DM, Meemon N, Lawpoolsri S, Singhasivanon P, Sattabongkot J, et al. Ownership and utilization of bed nets and reasons for use or non-use of bed nets among community members at risk of malaria along the Thai-Myanmar border. Malar J. 2021;20(1):305. doi: 10.1186/s12936-021-03837-5 34229653 PMC8259116

[32] Nkum CB, Ateudjieu J, Nanfak A, Tchio-Nighie KH, Mbiaketcha Nzinnou SI, Guenou E, et al. Long-Lasting Insecticidal Net Ownership and Use in Mogode Health District, Cameroon. Cureus. 2024;16(4):e57819. doi: 10.7759/cureus.57819 38721166 PMC11078082

[33] Nwachukwu CA, Anorue LI, Ajaero ID. Rural-Urban Differentials in Access to Behaviour Change Communication and Use of Long-Lasting Insecticide-Treated Nets and Artemisinin-Based Combination Therapy in Southeast Nigeria. Ethiopian Journal of Health Sciences. 2022;32(1). 10.4314/ejhs.v32i1.7 PMC8864383 35250217

[34] Konlan KD, Kossi Vivor N, Gegefe I, Hayford L. Factors associated with ownership and utilization of insecticide treated nets among children under five years in sub-Saharan Africa. BMC Public Health. 2022;22(1):940. doi: 10.1186/s12889-022-13347-x 35538524 PMC9092763

[35] Klu D, Aberese-Ako M, Manyeh AK, Immurana M, Doegah P, Dalaba M, et al. Mixed effect analysis of factors influencing the use of insecticides treated bed nets among pregnant women in Ghana: evidence from the 2019 Malaria Indicator Survey. BMC Pregnancy Childbirth. 2022;22(1):258. doi: 10.1186/s12884-022-04586-2 35346098 PMC8958761

[36] Nthambi M, Lembo T, Hanley N. Understanding decision-making and household health production in human and livestock systems within Sub-Saharan Africa: A review. Journal Title Abbreviation Needed. 2023;36(1):1–10.

[37] Moukénet A, Richardson S, Moundiné K, Laoukolé J, Ngarasta N, Seck I. Knowledge and practices surrounding malaria and LLIN use among Arab, Dazagada and Fulani pastoral nomads in Chad. PLoS One. 2022;17(4):e0266900. doi: 10.1371/journal.pone.0266900 35421160 PMC9009653

[38] Aung PL, Win KM, Show KL. Utilization of insecticide-treated bed nets among pregnant women in Myanmar-analysis of the 2015-2016 Demographic and Health Survey. PLoS One. 2022;17(3):e0265262. doi: 10.1371/journal.pone.0265262 35271668 PMC8912190

[39] Min KT, Maung TM, Oo MM, Oo T, Lin Z, Thi A, et al. Utilization of insecticide-treated bed nets and care-seeking for fever and its associated socio-demographic and geographical factors among under-five children in different regions: evidence from the Myanmar Demographic and Health Survey, 2015-2016. Malar J. 2020;19(1):7. doi: 10.1186/s12936-019-3088-0 31906965 PMC6945537

[40] Haileselassie W, Adam R, Habtemichael M, David RE, Solomon N, Workineh S, et al. Socio-demographic and economic inequity in the use of insecticide-treated bed nets during pregnancy: a survey-based case study of four sub-Saharan African countries with a high burden of malaria. Arch Public Health. 2023;81(1):64. doi: 10.1186/s13690-023-01075-6 37085893 PMC10122400

[41] Doe PF, Druye AA, Azu TD, Boso CM, Commey IT, Agyare DF, et al. Ownership and usage of insecticide-treated nets in Ghana: a scoping review of facilitators and barriers. Malar J. 2024;23(1):238. doi: 10.1186/s12936-024-05072-0 39127692 PMC11316354

[42] Acharya D, Adhikari R, Kreps GL, Wagle BP, Sharma S. An Association between the Mosquito Nets and the Wealth Status: Public Health Promotion Planning and Intervention. Prithvi Acad J. 2023;59–73. doi: 10.3126/paj.v6i1.54613

[43] Hambisa MT, Debela T, Dessie Y, Gobena T. Long lasting insecticidal net use and its associated factors in Limmu Seka District, South West Ethiopia. BMC Public Health. 2018;18(1):124. doi: 10.1186/s12889-018-5022-8 29321016 PMC5764020

[44] Seyoum D, Speybroeck N, Duchateau L, Brandt P, Rosas-Aguirre A. Long-Lasting Insecticide Net Ownership, Access and Use in Southwest Ethiopia: A Community-Based Cross-Sectional Study. Int J Environ Res Public Health. 2017;14(11):1312. doi: 10.3390/ijerph14111312 29077052 PMC5707951

[45] Ng’ang’a PN, Aduogo P, Mutero CM. Strengthening community and stakeholder participation in the implementation of integrated vector management for malaria control in western Kenya: a case study. Malaria Journal. 2021;20(1):155. doi: 10.1186/s12936-021-03692-4 33740983 PMC7977174

[46] Ahorlu CS, Adongo P, Koenker H, Zigirumugabe S, Sika-Bright S, Koka E, et al. Understanding the gap between access and use: a qualitative study on barriers and facilitators to insecticide-treated net use in Ghana. Malar J. 2019;18(1):417. doi: 10.1186/s12936-019-3051-0 31831004 PMC6909499

[47] Polec LA, Petkovic J, Welch V, Ueffing E, Ghogomu ET, Pardo JP, et al. Strategies to Increase the Ownership and Use of Insecticide‐Treated Bednets to Prevent Malaria. Campbell Systematic Reviews. 2015;11(1):1–127. doi: 10.4073/csr.2015.17 PMC7025867 25822171

[48] Chuma J, Okungu V, Ntwiga J, Molyneux C. Towards achieving Abuja targets: identifying and addressing barriers to access and use of insecticides treated nets among the poorest populations in Kenya. BMC Public Health. 2010;10:137. doi: 10.1186/1471-2458-10-137 20233413 PMC2847543

[49] Atieli HE, Zhou G, Afrane Y, Lee M-C, Mwanzo I, Githeko AK, et al. Insecticide-treated net (ITN) ownership, usage, and malaria transmission in the highlands of western Kenya. Parasit Vectors. 2011;4:113. doi: 10.1186/1756-3305-4-113 21682919 PMC3135563

[50] Mugisha EK. Enhancing malaria and anemia management through community engagement and health education: Assessing the impact of local health programs on prevention, awareness, and treatment-seeking behavior. 2024.

[51] Chen L, Chen T, Lan T, Chen C, Pan J. The Contributions of Population Distribution, Healthcare Resourcing, and Transportation Infrastructure to Spatial Accessibility of Health Care. Inquiry. 2023;60:469580221146041. doi: 10.1177/00469580221146041 36629371 PMC9837279

[52] Tapera O. Determinants of long-lasting insecticidal net ownership and utilization in malaria transmission regions: evidence from Zimbabwe Demographic and Health Surveys. Malar J. 2019;18(1):278. doi: 10.1186/s12936-019-2912-x 31429761 PMC6701104

